# Evaluation of Thyroid Volume Normalisation in Female Patients with Hashimoto Thyroiditis: A 12-Month Comparative Study of Combined Supplements and Photobiomodulation Versus Supplementation Alone

**DOI:** 10.3390/biomedicines13071555

**Published:** 2025-06-25

**Authors:** Venera Berisha-Muharremi, Bernard Tahirbegolli, Ruth Phypers, Reem Hanna

**Affiliations:** 1 Faculty of Medicine, University of Prishtina, Bulevardi i Dëshmorëve nn, 10000 Prishtina, Kosovo; venera.berisha@uni-pr.edu; 2 Poliklinika Endomedica, Muharrem Fejza Str. Nr. 84, 10000 Prishtina, Kosovo; 3 Endocrinology Clinic, University Clinical Center of Kosovo, Lagja e Spitalit, 10000 Prishtina, Kosovo; 4 Department of Management of Health Institution and Services, Heimerer College, 10000 Prishtina, Kosovo; bernardtahirbegolli@gmail.com; 5 National Sports Medicine Centre, Lagjia e Spitalit nn, 10000 Prishtina, Kosovo; 6 Laser Medicine Centre, 134 Harley Street, London W1G 7JY, UK; ruth@lasermedicine.co.uk; 7 Department of Restorative Dental Sciences, UCL—Eastman Dental Institute, Medical Faculty, University College London, London WC1E 6DE, UK; 8 Department of Head and Neck Academic Centre, Integrated Research, Division of Surgery and Interventional Science, Medical Faculty, University College London, London W1W 7TY, UK; 9 Department of Surgical Sciences and Integrated Diagnostics, University of Genoa, Viale Benedetto XV, 16132 Genoa, Italy; 10 Dental Faculty, Royal College of Surgeons Ireland (RCSI), 121-122 St Stephen’s Green, Dublin 2, D02 H903 Dublin, Ireland

**Keywords:** Hashimoto thyroiditis, mitochondrial homeostasis, photobiomodulation, PBM, thyroid dysfunction, thyroid volume, ultrasonography

## Abstract

**Background/Objectives**: Hashimoto thyroiditis (HT) is an autoimmune disease affecting the thyroid, often leading to hypothyroidism, even in individuals with adequate iodine intake. Despite achieving biochemical euthyroidism through levothyroxine (LT4) therapy, many patients continue to experience persistent symptoms, likely due to ongoing thyroid autoimmunity. Photobiomodulation (PBM) has shown promise in treating autoimmune conditions, but its effect on thyroid volume (TV) remains unclear. This study aimed to assess the efficacy of PBM combined with supplements in restoring thyroid function and normalising TV compared to the use of supplements alone. **Methods:** Ninety-eight females aged 20–50 years old were divided into two groups: Group 1 received PBM and supplements and Group 2 received supplements only. The PBM parameters were as follows: 820 nm wavelength, 200 mW power, continuous mode, 20 s per point at 8 points (32 J/cm^2^ per point), twice weekly for three weeks. Both groups received vitamin D3 supplementation (if serum < 40 ng/dL) and 100 µg of oral selenium daily. **Results:** Ninety-seven participants completed the study (51 in Group 1, 46 in Group 2). Group 1 showed significantly greater improvements in TV normalisation and weight loss and reductions in BMI, waist/hip circumference, waist-to-hip ratio, TSH, anti-TPO, anti-TG, and LT4 dosage (*p* < 0.05). **Conclusions:** This study demonstrates that low-fluence PBM combined with supplements can effectively improve thyroid function, reduce TV, and enhance anthropometric and clinical outcomes in HT patients. The protocol holds potential for broader application and further validation in larger trials.

## 1. Introduction

Hashimoto thyroiditis (HT), also known as chronic autoimmune thyroiditis, is the most common autoimmune endocrine disorder and a leading cause of hypothyroidism in iodine-sufficient regions. It affects approximately 5% of the global population, with a notable female predominance and a peak incidence between the ages of 30 and 60, resulting in a female-to-male ratio of up to 10:1 [1,2]. Subclinical hypothyroidism due to HT may affect up to 10–15% of older women, often remaining undiagnosed [3]. The incidence of hypothyroidism has risen globally, a trend attributed to factors such as greater clinical awareness and advancements in diagnostic testing [1,4]. It occurs when the immune system mistakenly attacks the thyroid, leading to inflammation and damage. This condition is typically characterised by the presence of thyroid peroxidase antibodies (anti-TPO) and thyroglobulin antibodies (anti-TG) [5]. Chronic autoimmune thyroiditis (CAT), the most common form of thyroid-specific autoimmune disorder, is marked by an autoimmune-inflammatory response and lymphocytic infiltration of thyroid follicles [6].

A characteristic feature of HT in many patients is an increased thyroid volume (TV), particularly in the early or subclinical stages of the disease.

### 1.1. Aetiopathogenesis of High TV in HT

The pathogenesis of increased TV in HT is multifactorial. Ongoing research into the molecular and cellular mechanisms of HT continues to shed light on the complex interactions between immune cells, pro-inflammatory cytokines, and thyroid epithelial tissue. These studies aim to better elucidate how many interactions drive thyroid tissue remodelling, fibrosis, immune-mediated inflammation, and lymphocytic infiltration [7,8,9,10]. These processes lead to the characteristic enlargement of the thyroid gland in affected individuals’ development of high TV and disease progression [11].

In HT, the immune system mounts an autoimmune response against thyroid antigens, leading to chronic inflammation, predominantly mediated by autoreactive T lymphocytes and the production of anti-thyroid peroxidase (anti-TPO) and anti-thyroglobulin (anti-TG) antibodies [12].

This immune activity results in extensive lymphocytic infiltration of the thyroid follicles, contributing to glandular remodelling and enlargement of the thyroid gland [7,8,9]. Infiltrating lymphocytes, including B-cells and T-cells, produce inflammatory cytokines such as TNF-α, IL-6, IL-1β, and IFN-γ, which exacerbate tissue damage and attract more immune cells, further inflaming the thyroid and causing it to swell [13].

There is evidence to suggest that thyroid hormone levels may influence thyroid size in HT. For instance, patients with HT who have relatively high free triiodothyronine (FT3) and low free thyroxine (FT4) levels tend to have higher TV, possibly due to increased deiodinase activity converting thyroxine (T4) to triiodothyronine (T3) locally. This hormonal profile may reflect a compensatory mechanism or altered thyroid metabolism promoting glandular hypertrophy [14].

Despite the increase in TV, thyroid function can be variable, with early stages potentially leading to hyperthyroidism as the thyroid compensates, followed by hypothyroidism as tissue destruction impairs hormone production. The impact of high TV complicates clinical management, as large glands can make assessment and treatment decisions challenging [12,15].

### 1.2. TV Changes in HT

In HT, changes in TV are closely linked to the disease stage, immune damage, and compensatory mechanisms of the thyroid. In the early stages, the thyroid may enlarge due to inflammation and lymphocytic infiltration, while in the later stages, fibrosis and atrophy lead to a reduction in size [16]. Smaller thyroid volumes are often associated with a more advanced disease and greater thyroid dysfunction.

A common ultrasound finding in HT is a hypoechogenic pattern, indicating inflammation and lymphocyte infiltration, which can precede the detection of anti-TPO and anti-TG. These ultrasound changes serve as early indicators of thyroid dysfunction. TV variability in HT patients is influenced by disease progression, with an initial increase in volume due to inflammation, followed by a decrease, as fibrosis and atrophy set in during the advanced stages of the disease [17,18]. It is noteworthy that there is gender difference in the level of TV in HT individuals. The higher TV in females with HT is likely due to a combination of hormonal influences (pregnancy and postpartum changes) [19], stronger immune responses [20], genetic factors [21], and gender-specific immune regulation [22], all of which contribute to the increased severity of autoimmune thyroid conditions in women.

### 1.3. High-Resolution Ultrasonography (USG) in Thyroid Assessment

The thyroid gland, when affected by HT, often exhibits structural changes that can be detected via imaging techniques such as ultrasonography [23]. High-resolution ultrasound continues to be the most effective and widely employed imaging technique for the evaluation of thyroid abnormalities [24]. Its non-invasive nature, ease of use, and cost-effectiveness make high-resolution ultrasound a valuable tool for assessing TV and identifying structural changes in thyroid disease. While it cannot assess thyroid function directly, ultrasound remains the gold standard for thyroid gland imaging, particularly in the context of goitre assessment and TV measurement.

In recent decades, the WHO has officially recognised sonography as the primary diagnostic tool for assessing goitre and calculating TV [25]. High-resolution ultrasound allows for accurate volume measurement by utilising specific mathematical formulas that consider the dimensions of the thyroid lobes, providing a detailed understanding of the gland’s size and morphology. This is particularly important for detecting subtle changes in TV that may be indicative of early thyroid disease, such as HT [26].

### 1.4. Role of Oral Supplements in Normalising TV

#### 1.4.1. Selenium Supplementation and TV

A systematic review and meta-analysis of randomised controlled trials (RCTs) showed that selenium supplementation significantly reduced anti-TPO and malondialdehyde levels in patients with HT. However, no significant changes were observed in TV [27]. Another systematic review and meta-analysis reported that selenium supplementation was associated with a significant reduction in anti-TPO and an improvement in mood and well-being. Nevertheless, changes in the TV were either unaltered or unreported [28]. Combined supplementation with myo-inositol and selenium has demonstrated a significant decrease in TV, alongside improvements in thyroid function and quality of life [29,30].

#### 1.4.2. Vitamin D and TV

A study conducted by Bozkurt et al., 2013 [31] demonstrated that serum 25-hydroxyvitamin D (25OHD) levels were significantly lower in HT patients compared to the controls. Moreover, 25OHO deficiency correlated with the duration of HT, TV, and antibody levels, suggesting a potential role of vitamin D in the development and progression of the disease. Deficiency in vitamin D is associated with increased TV and higher antibody levels in HT patients, suggesting that maintaining adequate vitamin D levels may be beneficial [31].

### 1.5. Photobiomodulation (PBM) Therapy in Reducing TV

PBM therapy has expanded into a diverse range of clinical applications to address multiple health conditions, which benefit from tissue regeneration [32,33,34,35], wound healing [36,37], the reduction in inflammation [38], and pain management [39,40,41].

With regard to HT, which is an autoimmune condition leading to chronic inflammation of the thyroid gland and a subsequent increase in TV, PBM therapy is researched for its potential benefits in reducing inflammation, improving thyroid function [42], and its potential to affect the volume of the thyroid gland.

#### 1.5.1. Reduction in Inflammation

PBM therapy is believed to have anti-inflammatory effects. Since HT involves chronic inflammation of the thyroid [13], PBM could reduce this inflammation, which might lead to a reduction in TV. By stimulating cellular processes that reduce oxidative stress (OS) and promote tissue repair, PBM might assist in helping to control the autoimmune response [43].

#### 1.5.2. Regulation of Immune System

Some studies suggest that PBM therapy may have immunomodulatory effects, potentially influencing the immune system’s activity in autoimmune diseases. For HT, this might mean that PBM could help modulate the immune response, possibly reducing the autoimmune attack on the thyroid gland [44,45].

#### 1.5.3. Improvement of Vascularisation

PBM therapy can enhance thyroid microcirculation and elevate serum concentrations of T3 and T4 in healthy animals. This improvement in vascularisation and hormone levels suggests that PBM may aid in tissue repair and regeneration [46].

#### 1.5.4. Normalising TV

Although research into PBM therapy’s direct effect on TV is limited, if inflammation and immune response are reduced, there may be a decrease in the size of the thyroid gland in individuals with HT [47,48,49]. The inflammation and autoimmune attacks in Hashimoto’s lead to thyroid gland enlargement (goitre) in many cases, and by addressing this inflammation, PBM may reduce thyroid size and volume and improve thyroid hormone levels [50].

#### 1.5.5. Thyroid Function

While PBM therapy might not directly impact thyroid hormone levels in all cases, by reducing inflammation and improving thyroid tissue health, it could potentially lead to improved thyroid function. This might result in better regulation of thyroid hormones, which are often disrupted in HT [42,48].

### 1.6. Study’s Rationale and Objectives

While the combination of PBM and supplements in the management of HT shows promising benefits, there remains limited clinical evidence to support its specific effectiveness in TV normalisation. This gap is largely due to heterogeneity in PBM dosimetry and treatment protocols, as well as the absence of long-term follow-up studies to assess the sustained effects of combined therapy versus supplementation alone. Taking these factors into account, our study aimed to evaluate the long-term effectiveness of PBM combined with supplements compared to supplements alone in achieving TV normalisation, supported by a large dataset. Additionally, we assessed other thyroid functionalities.

## 2. Materials and Methods

### 2.1. Study Design

Our non-randomised, open-label interventional clinical trial aimed to evaluate the effect of PBM and supplements on the thyroid volume and their effectiveness in restoring thyroid gland homeostasis in patients with HT compared to supplements alone. This study employed blinding strategies that included blinding both outcome adjudicators and data collectors. The procedure was conducted by a clinician with extensive experience in laser therapy and endocrinology.

This study was conducted at Poliklinika Endomedica, Prishtina, Kosovo. Subjects were enrolled between May 2022 and May 2024. The trial was registered at Poliklinika Endomedica on 25 March 2023, under registration number 02/2022. Ethical approval for the study was granted under approval number 2687.

#### 2.1.1. Population (P), Intervention (I), Comparison (C) and Outcome (O)—PICO

P: Female adults aged between 20 and 50 years old who were diagnosed with HT based on clinical and diagnostic criteria [51,52,53].

I: λ 820 nm laser PBM and supplements.

C: Supplements alone without PBM therapy.

O: Outcomes evaluated via ultrasound evaluation and biochemical and anthropometric measurements.

#### 2.1.2. Eligibility Criteria

##### Inclusion Criteria

Adult female subjects aged between 20 and 50 years old who were diagnosed with HT according to the following specific criteria: (1) high serum levels of thyroid autoantibodies anti-TPO and/or antiTG (anti-TPO ref. range < 34 IU/mL; antiTG ref range < 115 IU/mL) and (2) ultrasound findings of HT (GE Logiq V5 Ultrasound) [51,52,53].

##### Exclusion Criteria

- Patients with any known autoimmune diseases except HT or any other treatment except LT4.
- Adult females aged <20 and >50 years old.
- Adult males of any age group.
- Patients previously treated with radioiodine.
- Patients on immunosuppressants, immunostimulants, and any drug which could interfere with the production, transport and metabolism of thyroid hormones.
- Subjects with thyroid nodules or ectopic thyroid or thyroid hypoplasia.
- Hypothyroidism stemming from postpartum thyroiditis (up to 18 months after gestation).
- A history of Graves’ disease.
- Tracheal stenosis.
- Females who were pregnant or lactating.
- Subjects with a history of exposure to ionising irradiation and/or neoplasia in the cervical region.
- Patients with previous thyroid surgery.
- Patients with a serious illness (e.g., kidney and liver failure, cancer, stroke).

#### 2.1.3. Patient Cohort

After taking the eligibility criteria into consideration, 97 female subjects were recruited and unrandomised into two groups: Group 1 received PBM with supplements (*n* = 51) and Group 2 received supplements alone (*n* = 46).

The appropriate dose of LT4 replacement was determined by the endocrinologist for each patient before entering the trial and every three months. Each patient was given advice to avoid gluten and foods containing sugar.

Patients with a serum level of vitamin D3 < 40 ng/dL received replacement, and all had daily intake of 100 µg of oral selenium.

### 2.2. Treatment Protocols

#### 2.2.1. Ultrasound

Ultrasound (GE Logiq V5, GE Healthcare, secured from Solingen, Germany) was employed to evaluate the TV at pre-treatment (T0) and at 3-month (T1), 6-month (T2), 9-month, (T3) and 12-month (T4) follow-up timepoints. The anatomical borders of the thyroid gland on the skin were defined by eight target points (four points on each lobe of thyroid gland) and were marked with a surgical pen at a distance of 1 cm apart from each other in every PBM session.

#### 2.2.2. TV Calculation

A standard protocol for a thyroid sonogram involves two-dimensional (2D) grey-scale imaging of the right and left lobes, isthmus, and, if present, the pyramidal lobe, using both sagittal and transverse views.

A comprehensive thyroid sonographic report should include details on the thyroid’s position, shape, size, composition, echogenicity, and vascular pattern [54,55].

The three linear dimensions that were measured for each lobe were the length (L) and anterior–posterior (A-P) diameters of each lobe, which were assessed in sagittal view, while the width (W) was measured in transverse view. Alternatively, the A-P dimension could be measured using a transverse image.

The volume (V) of each lobe can be automatically calculated, after recording the abovementioned three linear dimensions, using the ellipsoid equation with a correction factor: V (mL) = L (cm) × A-P (cm) × W (cm) × 0.523) [56,57].

#### 2.2.3. PBM Dosimetry and Treatment Protocol

In this study, we employed our PBM dosimetry protocol described in a study published in 2023 [42]. Table 1 shows the laser device specifications, the study’s laser parameters, and the treatment protocols. A single laser probe (Omega XP, Omega Laser Systems Limited, Essex, UK) was employed, delivering a photonic energy of 820 nm at a therapeutic power output of 200 mW (measured with a PM160T power meter, Newton, NJ, USA) in continuous-emission mode. The irradiation time per point was 20 s. The total number of irradiation points was eight. The laser probe was held in contact and at 90° in relation to the target tissue, delivering a fluence of 32 J/cm^2^ per point, where a total fluence of 256 J/cm^2^/session (160 s/session) was delivered over the thyroid. The treatment protocol was a total of six sessions based on a frequency of twice a week (excluding weekends) for three consecutive weeks.

### 2.3. Outcomes Measures

#### 2.3.1. Primary Outcomes Measurement

The primary outcome was to evaluate the TV changes after PBM + supplements (Group 1) vs. supplements alone (Group 2) in terms of normalising TV over the follow-up period.

#### 2.3.2. Secondary Outcomes Measurement

The secondary outcomes were the improvement of thyroid gland functions and weight management in terms of a reduction in TSH levels, an increase in FT4 levels, a reduction in LT4 dose required for substitution, and reductions in body mass index (BMI) and waist and hip circumferences (waist-to-hip ratio calculated by dividing the waist circumference by the hip circumference) in Group 1 (PBM + supplements) vs. Group 2 (supplements alone).

### 2.4. Assessment Tools

#### 2.4.1. Ultrasound Measurements

Ultrasound calculation of the thyroid volume (GE Logiq V5, GE Healthcare, secured from Solingen, Germany) was employed to evaluate the TV over the period of the follow-up timepoints T1–T4 compared to T0 for both groups.

#### 2.4.2. Biochemical Measurement

The serum levels of TSH, FT4, FT3, antiTPO, and antiTG (Electro ChemiLuminescence technology for the immunoassay, Cobas e 411 Roche-Hitachi Analyzer, Hitachi High Technologies Corporation 1-24-14 Nishi-Shinbashi, Minato-ku, Tokyo 105-8717 Japan [58]) were measured at T0 and at the T1-T4 follow-up timepoints.

#### 2.4.3. Lifestyle Factors

Weight (kg), height (m), waist circumference (cm), hip circumference (cm), calculated BMI (weight (kg) per height (m^2^), and waist/hip ratio measurements were all evaluated at the T1-T4 follow-up timepoints.

### 2.5. Statistical Analysis

The sample size was calculated post hoc with the G-power programme, using the ANOVA axis, for 5 repeated measurements. It was estimated that the research sample, with 97 participants in total, for two groups with five measurements, with an effect size f of 0.25 and 0.05 α, had a power of 0.88 1-β.

The IBM SPSS v21.0 package programme was used to examine the data. Continuous variables were described using the mean and standard deviation (SD) or the median and interquartile range (IQR), whereas categorical variables were summarised using the frequency (n) and percentage (%).

The General Linear Model (GLM) repeated-measurement analysis (F) and time*group interaction effect was used to assess the differences across the variables. Following significant main effects, Tukey’s Honestly Significant Difference (HSD) post hoc test was calculated to perform pairwise comparisons between group means. In our GLM analysis, we calculated Partial Eta Squared (η^2^p) to assess the effect size of each factor. The assumption of sphericity was tested using Mauchly’s test, and, in cases of violation, correction by Greenhouse–Geisser was considered. While the Friedman test was used for repeated measures, and the Kruskal–Wallis H test with Dunn–Bonferroni post hoc correction was used for group comparisons of non-normally distributed variables. A *p*-value of <0.05 was considered statistically significant.

## 3. Results

**Figure 1:**
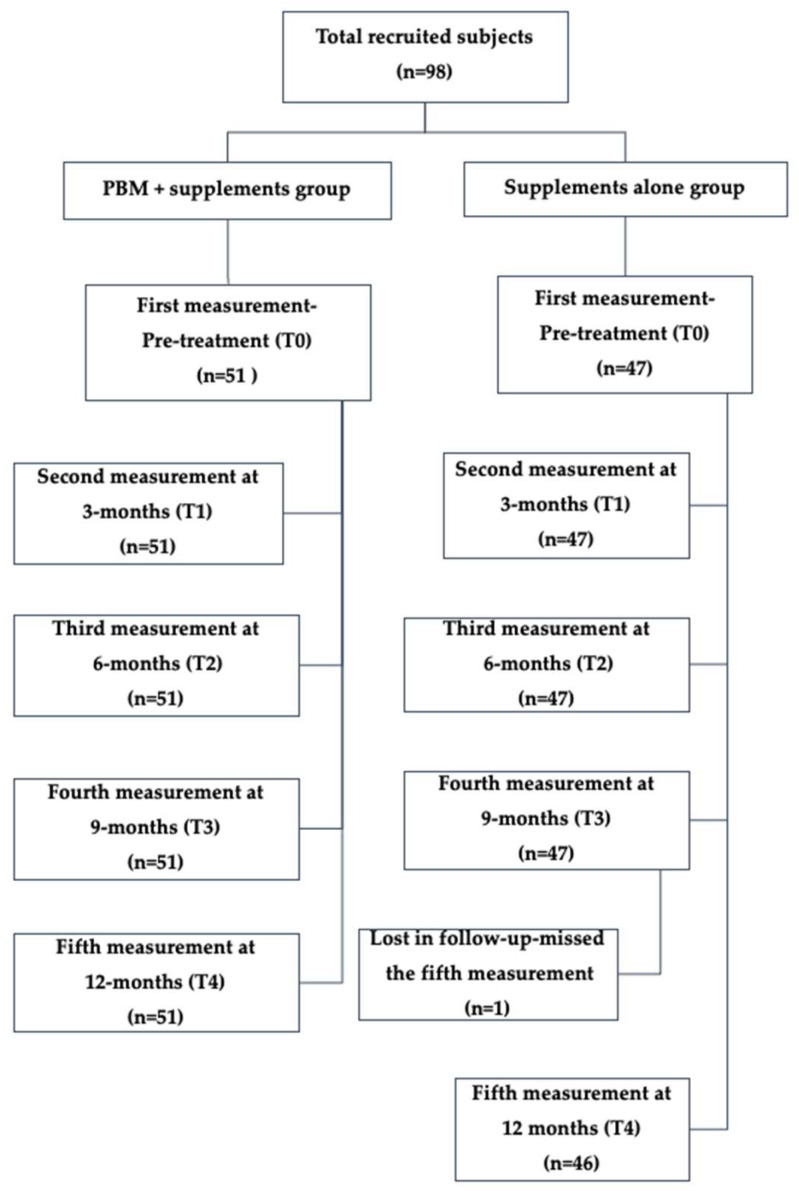
Cohort flow chart showing the study participants.

A total of 97 female subjects were included in the analysis, of whom 51 were treated with PBM therapy + supplements (Group 1) and 46 were treated with supplements alone (Group 2). The subject flow chart is illustrated in Figure 1.

TV measurements were obtained for all participants at timepoints T1, T2, and T3. At T4, one participant from the sham group was lost to follow-up, resulting in 46 subjects with available TV measurements at that timepoint. The statistical analysis was conducted for all the subjects who completed the TV for all five timepoints (T0–T4).

### 3.1. Demographic Characteristics

The mean age of the study subjects was 38.9 years old, with a BMI of 29.9 and a mean duration of treatment with LT4 of 48 months. The mean age of subjects in Group 1 was 38.9 years, with a body mass index (BMI) of 29.9 kg/m^2^ and a waist-to-hip ratio of 0.90. The median duration of levothyroxine (LT4) therapy in this group was 48.0 months. In comparison, the mean age of the subjects in Group 2 was 38.3 years old, with a BMI of 30.1 kg/m^2^, a waist-to-hip ratio of 0.90, and a median LT4 treatment duration of 45 months. These data are presented in Table 2.

### 3.2. Thyroid Parameters Measurements

A statistically significant difference was found in the values of TSH, FT3, FT4, anti-TPO, anti-TG, and TV during the 12 months of the PBM + supplements period compared to the initial values (*p* < 0.05). Meanwhile, in Group 2 (supplements alone), no statistically significant difference was found in the values of TSH, FT4, anti-TPO, anti-TG, and TV during the 12 months of the treatment period compared to the initial values (*p* > 0.05) (Table 3).

Analysed with post hoc pairwise analyses, it was found that the waist-to-hip ratio in T2–T4 (second to fifth measurement) was significantly lower compared to the first measurement prior to treatment (T0) in Group 1 (*p* < 0.05).

All other measurements were significantly lower than the first measured (T0) values of TSH in the Group 1 (*p* < 0.05). However, in Group 2, the fourth and fifth measurements at T3 and T4, respectively, were significantly lower than the second measurement (T2) (*p* < 0.05). The values of FT4 at T0 were significantly higher compared to all other measurements in Group 1 (*p* < 0.05). The mean values of the anti-TPO in the second to fifth measurements at T1 and T4, respectively, were significantly lower compared to T0 in Group 1 (*p* < 0.05).

The first measurement values of anti-TG in Group 1 were significantly higher compared to all other measurements (*p* < 0.05). Additionally, the third, fourth, and fifth measurements at T2, T3, and T4, respectively, were significantly lower compared to the second measurement (T1) of anti-TG values in the Group 1 (*p* < 0.05).

Post hoc pairwise analyses showed that the TV values in the second to fifth measurements were significantly lower compared to the first measurement (T0) in Group 1 (*p* < 0.05).

### 3.3. Time*Group Interaction Effect on Thyroid Variables

The repeated-measure ANOVA analysing the interaction effect between time and treatment group for TSH, FT3, FT4, anti-TPO, anti-TG, TV, and waist-to-hip ratio across the five measurement timepoints at T0-T4 showed a significant overall effect of PBM + supplements.

A repeated-measure ANOVA was conducted to examine the time*group effect of the waist-to-hip ratio over the five timepoint measurements (T0-T5). The results revealed a significant main effect of waist-to-hip ratio, F(df 2.280) = 15.866, *p* < 0.0001, with a moderate effect size (η^2^p = 0.143). Greenhouse–Geisser correction was applied due to a violation of sphericity.

There was a statistically significant difference in the repeated-measure ANOVA test for the time*group effect of the TSH, FT3, FT4, anti-TPO, and anti-TG values (*p* < 0.05) (Table 4). A repeated-measure ANOVA for the time*group interaction effect of the thyroid volume over the five timepoint measurements at T0-T4 revealed a significant main effect of PBM treatment, F (1.471) = 13.823, *p* < 0.0001, with a moderate effect size (η^2^p = 0.127).

### 3.4. Thyroid-Stimulating Hormone (TSH) Outcomes

**Figure 2:**
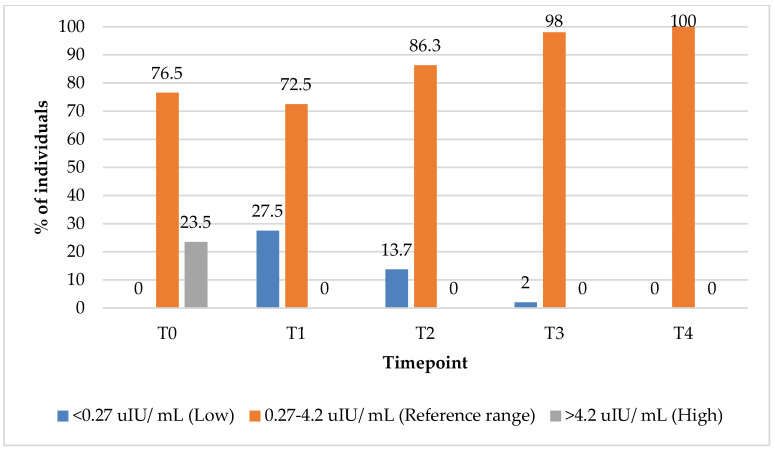
Thyroid-stimulating hormone (TSH) values over time among PBM + supplements group participants. Reference range: 0.27–4.2 uIU/ mL; low (below reference range): <0.27 uIU/ mL; high (above reference range): >4.2 uIU/ mL.

In the group treated with PBM + supplements, the percentage of subjects with TSH values within the reference range increased from 76.5% at T0 to 100% at 12 months (T4) (Figure 2). Concurrently, the percentage of subjects with elevated TSH levels declined from 23% at baseline (T0) to 0% by the 3-month (T1) follow-up timepoint, indicating an early and sustained normalisation of the thyroid function markers following the intervention.

**Figure 3:**
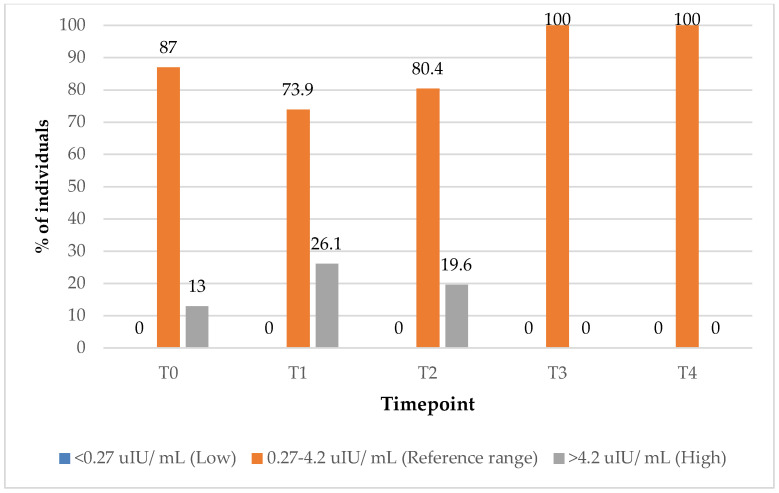
TSH values over time among participants treated with supplements alone. Reference range: 0.27–4.2 uIU/ mL; low (below reference range): <0.27 uIU/ mL; high (above reference range): >4.2 uIU/ mL.

In the group treated with supplements alone, the proportion of subjects with TSH values within the reference range increased from 87% at baseline (T0) to 100% at the 9-month (T3) follow-up timepoint (Figure 3). Interestingly, the percentage of subjects with elevated TSH values initially increased from 13% baseline (T0) to 26% at the 3-month (T1) follow-up timepoint, before subsequently declining to 0% by the 9-month mark, suggesting a delayed but complete normalisation of TSH levels over the course of treatment.

### 3.5. Thyroxine-FT4 Outcomes

**Figure 4:**
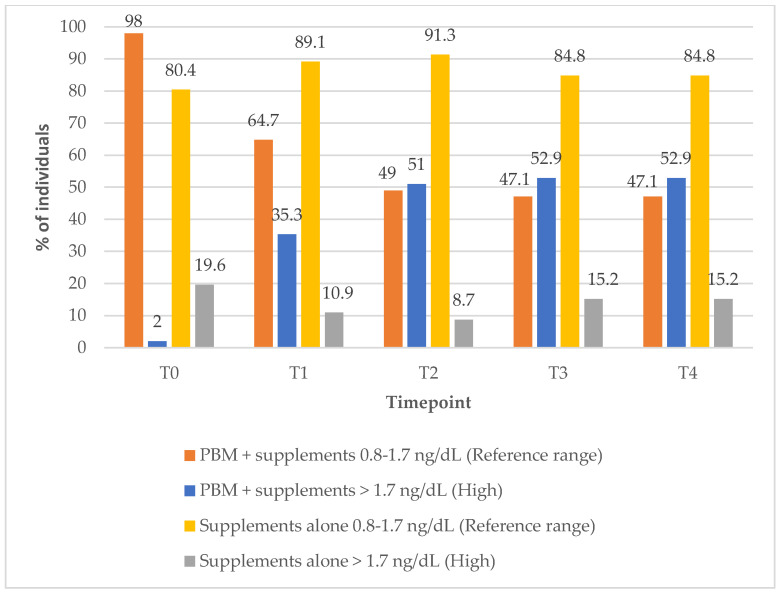
Thyroxine-FT4 values over time according to study group. Reference range: 0.8–1.7 ng/dL; high (above reference range): >1.7 ng/dL.

In Group 1, the proportion of the subjects with FT4 levels within the reference range decreased markedly over the course of the treatment, from 98% at baseline to 47% at the 12-month (T4) follow-up timepoint (Figure 4). In contrast, Group 2 showed a modest improvement, with the percentage of subjects within the reference FT4 range increasing from 80% at baseline to 85% at 12-month follow-up timepoint, suggesting differing effects on FT4 regulation between the two treatment approaches (PBM + supplements vs. supplements alone) (Figure 4).

**Figure 5:**
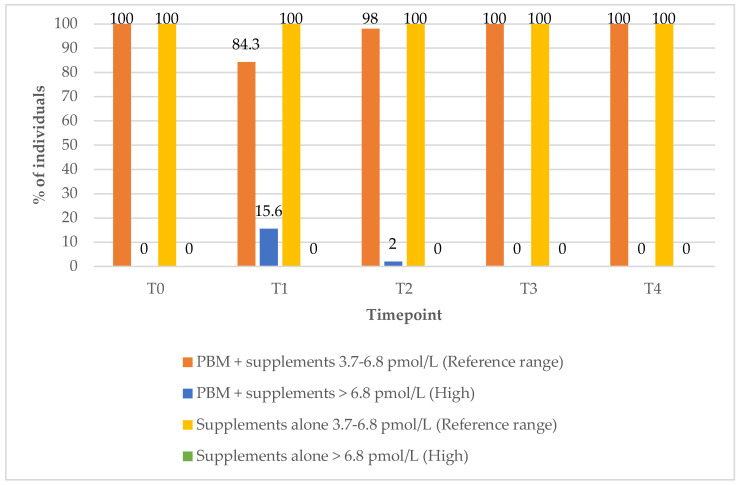
FT3 values over time according to study group. Reference range: 3.7–6.8 pmol/L; high (above reference range): >6.8 pmol/L.

Interestingly, there were no changes in the percentage of those within the FT3 reference range values in any of the study groups over the 12-month post-treatment period (Figure 5).

### 3.6. Free Triiodothyronine (FT3) Outcomes

**Figure 6:**
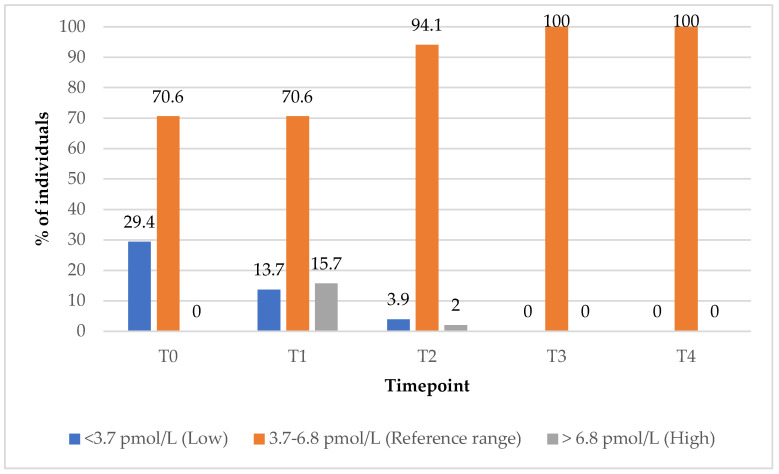
FT3 values over time among Group 1. Reference range: 3.7–6.8 pmol/L; low (below reference range): <3.7 pmol/L; high (above reference range): >6.8 pmol/L.

In the group treated with PBM + supplements (Group 1), the proportion of subjects with FT3 values within the reference range increased from 71% at baseline (T0) to 100% at the 9-month (T3) follow-up timepoint (Figure 6). The percentage of subjects with elevated FT3 levels rose from 0% at baseline (T0) to 16% at the 3-month (T1) follow-up timepoint, before returning to 0% by the 9-month timepoint (T3), indicating a transient increase followed by normalisation within the reference range (Figure 6).

**Figure 7:**
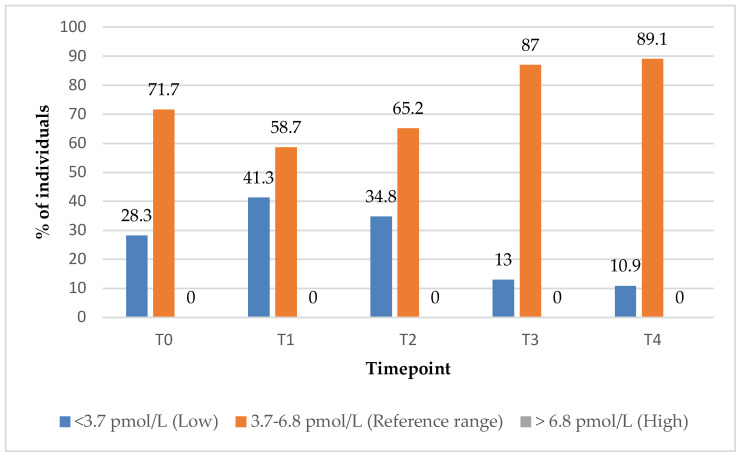
FT3 values over time among participants treated with supplements alone. Reference range: 3.7–6.8 pmol/L; low (below reference range): <3.7 pmol/L; high (above reference range): >6.8 pmol/L.

In the group treated with supplements alone (Group 2), the proportion of subjects with FT3 values within the reference range increased from 72% at baseline (T0) to 89% at the 12-month (T4) follow-up timepoint (Figure 7).

### 3.7. Anti-Thyroid Peroxide (Anti-TPO) Antibodies Outcomes

**Figure 8:**
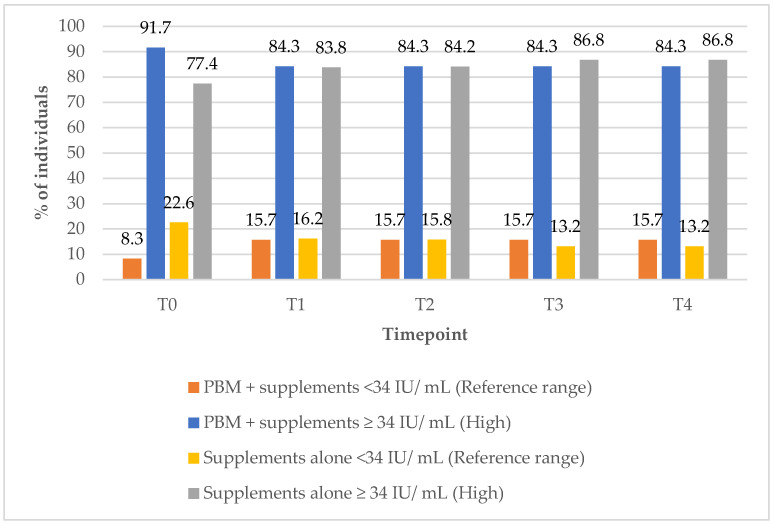
Anti-TPO values over time according to study group. Reference range: <34 IU/ mL; high (above reference range): ≥34 IU/ mL.

In Group 1, the proportion of subjects with anti-TPO antibody levels within the reference range increased from 8% at baseline (T0) to 16% at the 12-month (T4) follow-up timepoint (Figure 8). In contrast, Group 2 showed a decline in the percentage of subjects with reference range anti-TPO values, from 23% at baseline (T0) to 13% at the 12-month (T4) follow-up timepoint. All these data are illustrated in Figure 8.

### 3.8. Anti-Thyroglobulin (Anti-TG) Antibodies Outcomes

**Figure 9:**
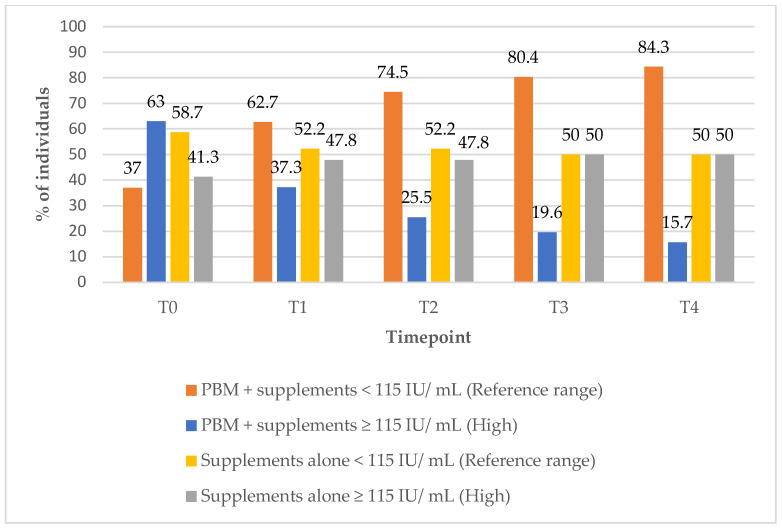
Anti-TG values over time according to study group. Reference range: <115 IU/ mL; high (above reference range): ≥115 IU/ mL.

In Group 1, the proportion of subjects with anti-TG antibody levels within the reference range increased significantly from 37% at baseline (T0) to 84% at the 12-month (T4) follow-up timepoint (Figure 9). In contrast, Group 2 demonstrated a decrease in the percentage of subjects with anti-TG levels within the reference range over the same period, from 59% to 50%, suggesting a more favourable immunological response in the combined treatment groups (Figure 9).

### 3.9. Thyroid Volume Normalisation Outcomes

**Figure 10:**
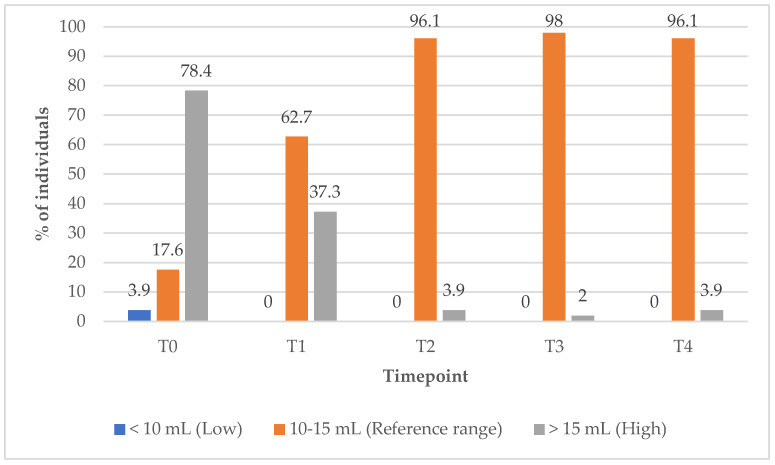
Thyroid volume (TV) values over time among PBM + supplements group participants. Reference range for adult women’s thyroid volume: 10–15 mL; low (below reference range): <10 mL; high (above reference range): >15 mL.

In Group 1, the proportion of subjects with TV within the reference range increased markedly from 17% at baseline (T0) to 96.1% at the 12-month (T4) follow-up timepoint (Figure 10). Concurrently, the percentage of subjects with elevated TV decreased from 78% at baseline (T0) to just 2% at the 9-month (T3) follow-up timepoint, indicating substantial improvements in thyroid size over the course of treatment (Figure 10).

**Figure 11:**
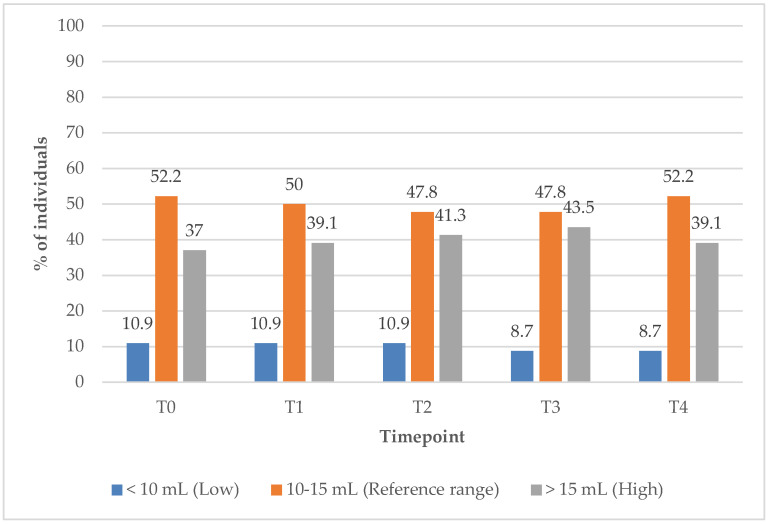
Thyroid volume values over time among participants treated with supplements alone. Reference range for adult women’s thyroid volume: 10–15 mL; low (below reference range): <10 mL; high (above reference range): >15 mL.

In Group 2, the proportion of subjects with TV within the reference range remained unchanged at 52.2% from baseline (T0) to the 12-month (T4) follow-up timepoint (Figure 11). Meanwhile, the percentage of individuals with elevated TV increased slightly, from 37% at baseline to 39% at T1, suggesting minimal improvement in TV under the supplements only treatment.

## 4. Discussion

Hypothyroidism is a systemic hypometabolic disorder characterised by thyroid hormone deficiency and is closely associated with OS. The cyclic adenosine monophosphate (cAMP)/protein kinase A (PKA) signalling pathway plays a critical role in regulating thyroid hormone secretion and mitigating OS. TV is a clinically relevant marker of disease activity and progression.

Our main finding indicated that PBM combined with supplements (Group1) resulted in statistically significant improvements in multiple thyroid-related parameters, including TV, TSH, FT3, FT4, and anti-TPO, among female subjects. Compared to Group 2, Group 1 demonstrated significantly greater improvement across the 12-month follow-up period in TSH, FT3, FT4, anti-TPO, anti-TG, TV, and waist-to-hip ratio.

Notably, the proportion of subjects with elevated TV in Group 1 decreased markedly from 78% at T0 to 37% at T1, and further to 3.9% at T2. In contrast, in Group 2, between 37% and 43% of subjects continued to present with elevated TV throughout the same follow-up period, highlighting the superior efficacy of the combined treatment approach, PBM and supplements, in reducing thyroid hypertrophy.

Combined treatment with PBM and supplements may exert synergistic effects on thyroid health. Evidence from previous studies suggests that combined therapy leads to greater reductions in TV, improved echogenicity, and more pronounced improvements in clinical symptoms and thyroid autoantibody levels compared to either intervention alone [55,59]. From a mechanistic standpoint, PBM primarily exerts local effects, targeting inflammation, immune modulation, and tissue remodelling within the thyroid gland. In contrast, treatment with supplements alone contributes to systemic immune regulation and micronutrient repletion, addressing underlying deficiencies such as selenium that are often implicated in autoimmune thyroid disorders [54,60,61].

PBM tends to produce faster local responses including reduced thyroid inflammation and volume, while supplements may require a longer duration to yield systemic and morphological changes. Importantly, PBM is non-invasive, generally well-tolerated, and associated with a low risk of adverse effects. Current evidence indicates that combined therapy does not increase the incidence of adverse events and may offer enhanced efficacy without compromising safety [59]. Nevertheless, to maximise therapeutic outcomes, careful consideration must be given to the optimal parameters, including irradiance, frequency and duration of treatment.

## 5. Conclusions and Future Directions

To the best of the authors’ knowledge, this is the first long-term follow-up study to demonstrate that combining PBM with supplements may offer a more comprehensive treatment strategy for normalising TV, preserving thyroid structure and function, and potentially delaying or preventing the progression of hypothyroidism, compared to supplements alone. This study builds upon our initial 6-month observational research on the effects of PBM therapy in patients with HT [38]. Its primary strength lies in being the first to demonstrate the potential of PBM therapy to normalise TV in women with HT. Furthermore, we extended the follow-up period to one year to assess the sustained effects of the therapy not only on TV but also on thyroid function and antibody levels. A key limitation of our study, however, is the relatively small sample size. At this stage, the authors recommend that future RCTs adopt this study protocol with larger sample sizes and extended follow-up periods to validate these results and support the integration of combined therapy into HT management guidelines.

## Figures and Tables

**Table 1:** Photobiomodulation device specifications, dosimetry, and treatment protocol [42].

Device Specifications	Manufacturer	Omega XP, Laser Systems Limited, Essex, UK
	Emitters Type	GaAlAs diode laser
	Medical/laser class	3B
	Beam delivery system	Fibre
	Probe design	Single probe
	Beam profile	Gussian
Irradiation parameters	Wavelength	820 nm
	Therapeutic power output	200 mW
	Emission mode	Continuous (CW)
	Total energy per session (J)	32
	Number of irradiated points above thyroid	8
	Total irradiation time (s) per session	160
	Irradiation time (s) per point	20
	Fluence (J/cm^2^) per point	32
	Total fluence (J/cm^2^) per session	256
Treatment Protocol	Treatment frequency	Twice a week
	Treatment duration	Three consecutive weeks
	Total treatment sessions	6
	Treatment technique	Stationary application
	Light-tissue distance	Direct/in-contact with the skin

**Table 2:** Demographic characteristics of study groups.

Variable	Total (*n* = 97) Mean ± SD or Median (IQR)	Group 1 (*n* = 51) Mean ± SD or Median (IQR)	Group 2 (*n* = 46) Mean ± SD or Median (IQR)
Age (years)	38.9 ± 5.7	39.4 ± 5.7	38.3 ± 5.8
Duration of levothyroxine (LT4) treatment (months)	48.0 (24.0–84.0)	48.0 (24.0–118.0)	45.0 (21.0–60.0)
Body mass index (BMI) measurement (kg/m^2^)	29.9 ± 3.4	29.7 ± 3.6	30.1 ± 3.2
Waist-to-hip ratio measurement	0.9 ± 0.1	0.9 ± 0.1	0.9 ± 0.1

**Table 3:** Changes in thyroid parameters over time by study group.

Group	Parameters	Measurements					Statistical Significance
		First at Pre-Treatment (T0) Mean ± SD or Median (IQR)	Second at 3-Month Follow-Up (T1) Mean ± SD or Median (IQR)	Third at 6-Month Follow-Up (T2) Mean ± SD or Median (IQR)	Fourth at 9-Month Follow-Up (T3) Mean ± SD or Median (IQR)	Fifth at 12-Month Follow-Up (T4) Mean ± SD or Median (IQR)	
PBM + Supplements (Group 1)	Waist-to-Hip ratio	0.87 ± 0.07	0.85 ± 0.07	0.84 ± 0.09	0.83 ± 0.09	0.82 ± 0.09	F = 15.405; df = 2.452; *p* < 0.0001; η^2^p = 0.236
	TSH (uIU/mL)	3.19 ± 2.19	1.31 ± 0.98	1.27 ± 0.73	1.45 ± 0.48	1.40 ± 0.36	F = 31.372; df = 1.289; *p* < 0.0001; η^2^p = 0.386
	FT4 (ng/dL)	1.38 ± 0.25	2.42 ± 1.41	2.17 ± 1.02	1.94 ± 0.61	1.94 ± 0.61	F = 12.660; df = 1.853; *p* < 0.0001; η^2^p = 0.202
	FT3 (pmol/L)	3.87 ± 1.31	5.12 ± 1.50	5.10 ± 0.81	5.01 ± 0.49	5.01 ± 0.49	F = 16.193; df = 2.025; *p* < 0.0001; η^2^p = 0.245
	Anti-TPO (IU/mL)	353.00 (123.00–552.00)	100.40 (82.00–201.00)	90.30 (60.00–246.60)	88.00 (60.00–134.00)	88.00 (60.00–123.00)	X^2^ = 107.374; *p* < 0.0001;
	Anti-TG (IU/mL)	294.60 (39.57–444.75)	85.00 (21.75–221.77)	51.50 (22.30–122.10)	40.00 (18.50–104.25)	40.00 (18.50–99.77)	X^2^ = 92.299; *p* < 0.0001;
	TV (mL)	16.93 ± 3.24	15.01 ± 2.07	14.12 ± 1.48	13.78 ± 1.13	14.28 ± 4.23	F = 14.414, df = 1.437; *p* < 0.0001; η^2^p = 0.224
Supplements alone (Group 2)	Waist-to-Hip ratio	0.87 ± 0.08	0.88 ± 0.08	0.88 ± 0.08	0.88 ± 0.09	0.88 ± 0.09	F = 2.423; df = 1.515; *p* = 0.110; η^2^p = 0.051
	TSH (uIU/mL)	3.43 ± 3.18	3.47 ± 1.36	3.97 ± 4.33	2.50 ± 0.77	2.52 ± 0.74	F = 3.219; df = 1.980; *p* = 0.045; η^2^p = 0.067
	FT4 (ng/dL)	1.45 ± 0.33	1.38 ± 0.37	1.33 ± 0.28	1.45 ± 0.32	1.42 ± 0.33	F = 1.441; df = 2.642; *p* = 0.237; η^2^p = 0.031
	FT3 (pmol/L)	4.02 ± 0.95	3.73 ± 1.04	3.78 ± 0.82	4.24 ± 0.61	4.22 ± 0.52	F = 5.548; df = 2.704; *p* = 0.002; η^2^p = 0.110
	Anti-TPO (IU/mL)	266.75 (102.02–560.47)	153.50 (98.95–404.25)	171.85 (88.17–454.60)	174.50 (99.97–450.00)	174.50 (99.97–450.00)	X^2^ = 3.783; *p* = 0.436;
	Anti-TG (IU/mL)	73.25 (20.82–342.87)	112.00 (22.60–389.87)	105.85 (21.70–432.60)	111.85 (22.00–374.50)	107.15 (24.75–372.75)	X^2^ = 4.573; *p* = 0.334;
	TV (mL)	14.88 ± 3.24	14.84 ± 3.50	14.91 ± 3.59	15.03 ± 3.65	15.04 ± 3.69	F = 2.490; df = 1.496; *p* = 0.105; η^2^p = 0.052

Notes: A statistically significant difference (*p* < 0.05) was observed in the levels of TSH, FT3, FT4, anti-TPO, anti-TG, and thyroid volume over the 12-month PBM treatment period (T1–T4) compared to baseline (T0) values. In contrast, the conventional treatment group showed no statistically significant changes (*p* > 0.05) in TSH, FT4, anti-TPO, anti-TG, and thyroid volume over the same period. Abbreviations: T0: first measurement (baseline); T1: second measurement after 3 months; T2: third measurement after 6 months; T3: fourth measurement at 9 months; T4: fifth measurement after 12 months; TSH: thyroid-stimulating hormone; FT4: thyroxine; FT3: free triiodothyronine; anti-TPO: thyroid peroxidase antibody; anti-TG: antithyroglobulin antibodies; TV: thyroid volume; F: ANOVA; df: degree of freedom; η^2^p: Partial Eta Squared.

**Table 4:** Time*group interaction effect for thyroid parameters: ANOVA results.

Variable	Time*Group Interaction Effect
Waist-to-Hip ratio	F = 15.866; df = 2.280; *p* < 0.0001; η^2^p = 0.143
TSH (uIU/mL)	F = 6.740; df = 2.135; *p* = 0.001; η^2^p = 0.066
FT4 (ng/dL)	F = 12.208; df = 1.975; *p* < 0.0001; η^2^p = 0.114
FT3 (pmol/L)	F = 13.225; df = 2.437; *p* < 0.0001; η^2^p = 0.122
Anti-TPO (IU/mL)	F = 20.363; df = 1.360; *p* < 0.0001; η^2^p = 0.177
Anti-TG (IU/mL)	F = 12.984; df = 1.359; *p* <0.0001; η^2^p = 0.121
TV (mL)	F = 13.823; df = 1.471; *p* < 0.0001; η^2^p = 0.127

Notes: The repeated-measure ANOVA analysing the interaction effect between time and treatment group for TSH, FT3, FT4, anti-TPO, anti-TG, TV, and waist-to-hip ratio across five measurement points showed a significant overall effect of PBM treatment. Abbreviations: thyroid-stimulating hormone (TSH); thyroxine (FT4); free triiodothyronine (FT3); thyroid peroxidase antibody (anti-TPO); antithyroglobulin antibodies (anti-TG); F: ANOVA; df: degree of freedom; η2p: Partial Eta Squared.

## Data Availability

Data are contained within the article.
